# Students’ Perception of Online Versus Face-to-Face Learning: What Do the Healthcare Teachers Have to Know?

**DOI:** 10.7759/cureus.54217

**Published:** 2024-02-14

**Authors:** Ammar Ahmed Siddiqui, Malik Zain Ul Abideen, Saman Fatima, Muhammad Talal Khan, Syed W Gillani, Zeyad A Alrefai, Muhammad Waqar Hussain, Hassaan A Rathore

**Affiliations:** 1 Preventive Dental Sciences, University of Ha'il, Ha'il, SAU; 2 Dental Education & Research, Bakhtawar Amin Medical and Dental College, Multan, PAK; 3 Medical Education and Simulation, Bakhtawar Amin Medical and Dental College, Multan, PAK; 4 Dental Biomaterials, Bakhtawar Amin Medical and Dental College, Multan, PAK; 5 Clinical Pharmacy, Gulf Medical University, Ajman, ARE; 6 Management and Information Systems Development, University of Ha'il, Ha'il, SAU; 7 Prosthodontics, Bakhtawar Amin Medical and Dental College, Multan, PAK; 8 Pharmacy, Qatar University Health, Doha, QAT

**Keywords:** healthcare education, skills, pandemic, perception, knowledge, face to face teaching, e-learning

## Abstract

During the COVID-19 pandemic, educational institutions confronted the possibility of complete closure and took countermeasures by adapting e-learning platforms. The present cross-sectional study quantified the impact of the pandemic on medical education using a validated and reliable tool. The tool was used to explore the perceptions of 270 healthcare students about e-learning in comparison to traditional learning systems. Inferential statistics were employed using Pearson’s chi-squared test. It was found that e-learning was advantageous because of its location flexibility (46.1%) and the ease of access to study materials (46.5%). However, in-person learning was found to lead to an increase in knowledge (44.9%), clinical skills (52.7%), and social competencies (52.7%). The study concluded that while e-learning offers flexibility, traditional face-to-face teaching is deemed more effective for skill development and social interaction. Hence, e-learning should complement rather than replace traditional methods due to limitations in replicating clinical environments.

## Introduction

Effective teaching is a cardinal element of students’ prosperity. Teaching has been disrupted throughout the world since the outset of the COVID-19 pandemic, primarily affecting healthcare, medical, and dental institutions. To ensure patients’, students’, and teachers’ well-being during the pandemic, a suspension of face-to-face learning sessions and a shift to online classes took place worldwide [1]. Universities and medical and dental schools adapted to the e-learning approach to facilitate the uninterrupted delivery of knowledge to students.

Although universities and teaching institutions in developing countries lacked the strategies and infrastructure for online teaching. The e-learning approach became their mainstay for the provision of knowledge. E-learning is used to enhance the quality of education provided to students through information technology (IT) [2]. At present, various platforms for online teaching and learning are available, ranging from independent software solutions to university-owned modules and learning management systems [3,4]. A combination of online and conventional teaching methods was used for teaching undergraduate students throughout the pandemic [5,6]. Online learning helps students regulate the pace of their learning and monitor how they learn [7]. However, students in such a learning environment must be self-regulated and highly motivated to achieve their goals [8].^ ^Furthermore, the effectiveness of e-learning depends on a number of variables such as course content, access to facilities, usage of appropriate teaching methods, and assessment criteria.

E-learning has its own advantages and disadvantages similar to other teaching methods. Some of the advantages of e-learning are higher convenience, reduction in travel expenses, reduced air pollution due to lower traffic, and easier access to learning resources irrespective of the time and location [9,10]. Some of the disadvantages of e-learning are poor internet access, insufficient computer literacy, and poor internet connections. Flexibility of time may be a limitation of e-learning in cases where students lack self-discipline [5]. ^ ^Moreover, teachers, facilitators, and students reported facing numerous organizational, social, and technical challenges during online teaching and learning sessions throughout the pandemic [11,12].

A study conducted at a medical university in Jordan in 2021, revealed that the satisfaction rate with e-learning was quite low at 28.6% [13,14]. ^ ^In another study conducted at a medical university in the Middle East in 2019, half of the students considered online classes to be either equal to or superior to face-to-face learning [15]. Similarly, studies conducted in countries like Liberia and Egypt in 2021, suggested that students’ acceptance of e-learning was high [16]. The research findings indicated that the quality of the system, individuals' self-efficacy in computer use, and their proficiency with computers play significant roles in shaping the perceived ease of use of e-learning systems. Furthermore, factors such as the quality of information, enjoyment derived from the system, and accessibility were identified as contributors to both the perceived ease of use and perceived usefulness of e-learning systems [17].

Teachers are vital stakeholders in online and face-to-face teaching and learning [18]. They need to effectively implement online teaching and learning. Perceptions influence behavior; hence, it is critical to determine the effects of medical teachers’ perceptions on e-learning. Previous studies conducted in 2021, suggested that medical teachers prefer live/synchronous lectures over prerecorded lectures. According to them, these live sessions are more beneficial for postgraduate residents and trainees, and poor feedback from students is one of the drawbacks of online teaching. This poor feedback may be due to a lack of awareness, preparation, and training for online sessions [19]. In a study, it was found that 88% of faculty members agreed that possessing technological skills improves the educational value of the college staff's experience in offering online courses [20]. The aim of this study is to evaluate students’ perceptions regarding conventional face-to-face classes compared to online teaching sessions.

## Materials and methods

The research was carried out at the Institute of Dentistry, Bakhtawar Amin Medical and Dental College (BAMDC), located in Multan, Pakistan. The objective was to assess the effectiveness of both e-learning and face-to-face instructional sessions. Data were collected in the month of August and September 2021.

This study employed an observational approach with a cross-sectional design. The target population consisted of all the students from all the health colleges of BAMDC (Medical, Dental, Physiotherapy, and Allied health sciences). The combined student population across all health-related colleges amounted to 959 individuals. The target population was approached for data collection using an online link to Google Forms. This link was sent through the respective WhatsApp groups of each class. However, only 270 healthcare students participated in the study after providing informed consent with a response rate of 28%.

The inclusion criteria comprised undergraduate healthcare students who had experience with both online and face-to-face teaching and had provided consent by signing the informed consent form. Conversely, participants who declined to sign the informed consent form were excluded from the study.

Data collection

Data were collected through non-probability sampling by approaching all the targeted population. A questionnaire created using Google Forms was shared via email and WhatsApp groups. The present study used an existing questionnaire (which had already been validated and considered reliable) as the study tool [21]. The questionnaire consisted of four segments.

In the initial section, students were requested to furnish their demographic information (such as health facility, age group, gender, and year of study), indicate their proficiency in IT skills, and specify whether they had prior experience with online learning. The subsequent section prompted students to indicate their agreement or disagreement with various statements regarding the advantages and disadvantages of e-learning.

In the following segment, students utilized a Likert scale (ranging from 1 = extremely ineffective to 5 = extremely effective) to evaluate the efficacy of face-to-face and online learning in achieving learning objectives, including knowledge acquisition, clinical skill development, and social competencies. Additionally, students rated their level of engagement during classes on a scale from 1 (extremely inactive) to 5 (extremely active). In the final section, students provided their assessment of their level of satisfaction with e-learning experiences, utilizing a scale ranging from 1 (extremely unenjoyable) to 5 (extremely enjoyable).

Statistical analysis

The current study utilized categorical data, presented as n (%) and through figures. Data entry and analysis were conducted using Statistical Package for the Social Sciences version 21 (IBM Corp., Armonk, NY). Inferential statistics were applied, employing Pearson’s chi-squared test. Statistical significance was determined as a p-value of ≤ 0.05. Ethical clearance, referenced as IRB/905/21, was obtained from the Institutional Review Board of Bakhtawar Amin Medical and Dental College.

## Results

A total of 270 respondents filled out the questionnaire. The majority of the respondents were male. The demographics, as Table 1 shows, were categorized into six sections: health facility, age, gender, level of studies, IT skills, and previous e-learning experience. The majority of the respondents were from the medical college, followed by the dental college. The lowest responders were from Physiotherapy and Allied health sciences colleges. Most of the respondents were 21 to 23 years old. They were followed by 18 to 20-year-old respondents. The 24 to 26-year-old respondents were the lowest in number. Interestingly, a single participant from the 27 to 30-year-old age group participated in the study.

**Table 1:** Frequency table for nominal variables

Variables	Frequency (n)	Percentage %
Health Facility		
Medical college	145	53.70
Dental college	84	31.11
Physiotherapy	29	10.74
Allied health sciences	12	4.44
Age		
18-20	111	41.11
21-23	121	44.81
24-26	37	13.70
27-30	1	0.37
Gender		
Male	148	54.81
Female	122	45.19
Academic Year		
1st Year	60	22.22
2nd Year	85	31.48
3rd Year	70	25.93
4th Year	30	11.11
5th Year	21	7.78
Missing	4	1.48
IT Skills		
High	48	17.78
Moderate	152	56.30
Low	70	25.93
Missing	0	0.00
Previous E-learning Experience		
Yes	184	68.15
No	86	31.85

Furthermore, most of the respondents were second-year students, followed by third-year and first-year students. The lowest number of respondents were in their fourth and fifth academic years. In the demographic details section of the questionnaire, how the respondents rated their IT skills was interesting. The majority of the respondents rated their IT skills as moderate. Fewer respondents rated their IT skills as low, and only a fraction of the respondents were confident enough to mark their IT skills as high.

The latter part of the demographic details section questioned respondents about their previous e-learning experience. The majority of the respondents claimed to have previous experience. Only a few had no previous experience. Table 2 shows the level of significance of each question about the demographic details.

**Table 2:** Questions and demographic variables

Questions/Variables	Health Facility (P-value)	Age (P-value)	Gender (P-value)	Academic Year (P-value)	Level of IT Skills (P-value)	History of E-learning before pandemic (P-value)
Effectiveness of e-learning in terms of increasing knowledge	0.01**	0.49	0.35	0.24	0.03**	0.03**
Effectiveness of e-learning in terms of increasing clinical skills	0.39	0.46	0.01**	0.002**	0.09	0.03**
Effectiveness of e-learning in terms of social competencies	0.18	0.00**	0.17	0.004**	0.06	0.15
Effectiveness of face-to-face-learning in terms of increasing knowledge	0.08	0.06	0.50	0.27	0.48	0.13
Effectiveness of face-to-face-learning in terms of increasing clinical skills	0.17	0.21	0.18	0.50	0.03**	0.49
Effectiveness of face-to-face-learning in terms of increasing social competencies	0.001**	0.06	0.69	0.13	0.09	0.77
Activity during e-learning	0.17	0.006**	0.66	0.38	0.05*	0.22
Activity during traditional face to face-learning	0.09	0.13	0.02	0.85	0.36	0.07

The association between the health facility and level of IT skills and the effectiveness of e-learning in terms of increasing knowledge was significant (p < 0.05). The association between gender, academic year, and a previous history of e-learning and the effectiveness of e-learning in terms of increasing clinical skills was statistically significant (Table 2).

The effectiveness of e-learning in terms of increasing social competencies was statistically significant in the context of demographic details such as age and academic year. The effectiveness of face-to-face learning in terms of increasing clinical skills was statistically significant in the context of the IT skills of the respondents. The activities of students during e-learning in the context of demographic details such as age and level of IT skills were statistically significant. The activities of students during traditional face-to-face teaching in the context of gender were statistically significant (Table 2).

The effectiveness of e-learning in terms of increasing social competencies was rated, and it was found that the relationship of this with some variables (age and academic year) was significant. Similarly, the relationship between gender and the effectiveness of e-learning in terms of increasing clinical skills and between gender and activities during traditional face-to-face learning was found significant. Moreover, the relationship of health facilities with the effectiveness of e-learning in terms of increasing knowledge and the effectiveness of face-to-face learning in terms of increasing social competencies was found significant. The demographic variable year of study was found significant in relation to the effectiveness of e-learning in terms of increasing clinical skills. The relationship between IT skills and the effectiveness of face-to-face learning in terms of increasing knowledge was found significant. Previous e-learning experience had a significant relationship with the effectiveness of e-learning in terms of increasing knowledge and in terms of increasing clinical skills (Table 2).

**Figure 1:**
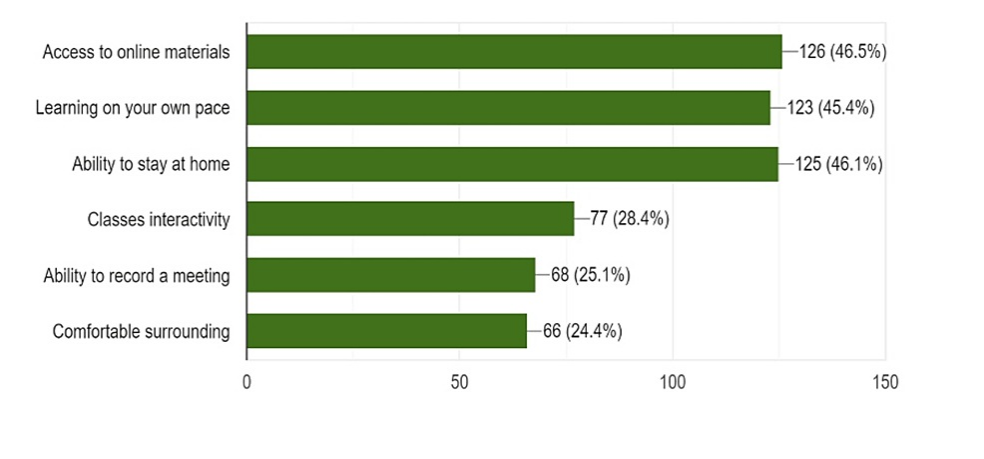
Advantages of e-learning

In response to a question related to the advantages and disadvantages of e-learning, most of the respondents rated access to online materials (n = 126, 46.5%) as the chief advantage, followed by the ability to stay at home (n = 1125, 46.1%) and the prospect of learning at their own pace (n = 123, 45.4%) (Figure 1).

**Figure 2:**
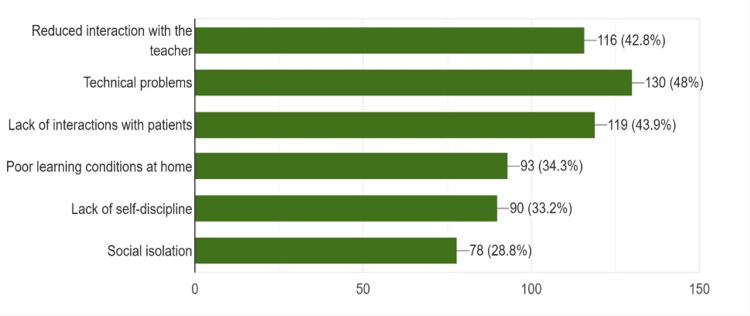
Disadvantages of e-learning

Most of the respondents rated technical problems (n = 130, 48%) as the chief disadvantage, followed by the inability to interact with patients (n = 119, 43.9%) and minimal interaction with teachers (n = 116, 42.8%) (Figure 2).

**Figure 3:**
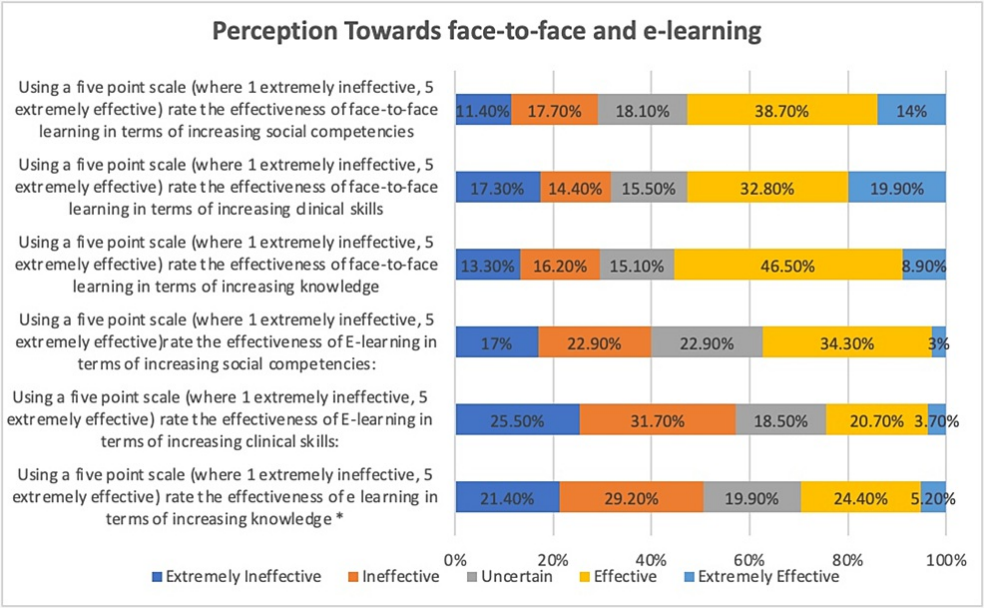
Comparison between face to face and e-learning Perception toward face-to-face and e-learning

The majority of the students did not consider e-learning more effective in terms of increasing knowledge. They considered it ineffective. However, a limited number of the students considered it effective (Figure 3).

**Figure 4:**
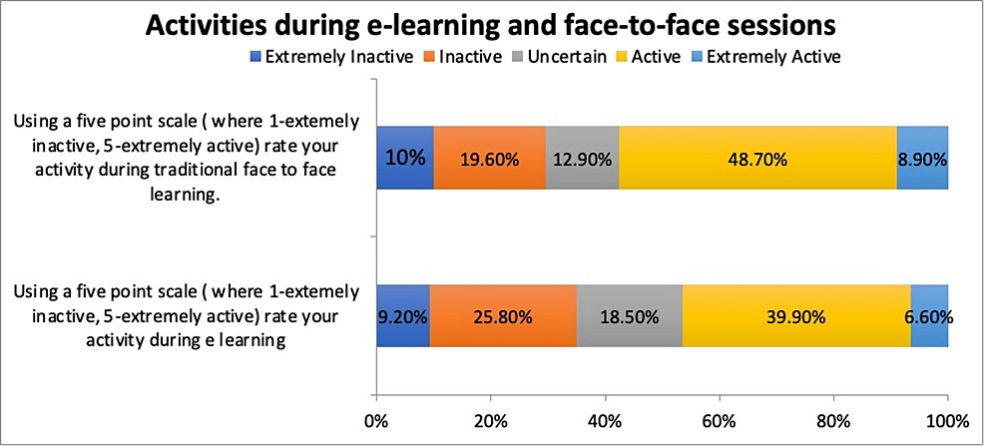
Activity during e-learning and face-to-face sessions

The majority of the respondents considered face-to-face learning more effective in improving clinical skills. However, fewer respondents believed that e-learning was a valuable resource for enhancing clinical proficiency, with a small number of responses describing it as effective (Figure 4).

Additionally, most respondents rated social adaptation to face-to-face learning as effective. In comparison, a limited number of respondents believe social adaptation to e-learning is effective. During traditional learning, most of the students claimed that they were active. Similarly, the participants who believed they were active during online were fewer (Figure 4).

**Figure 5:**
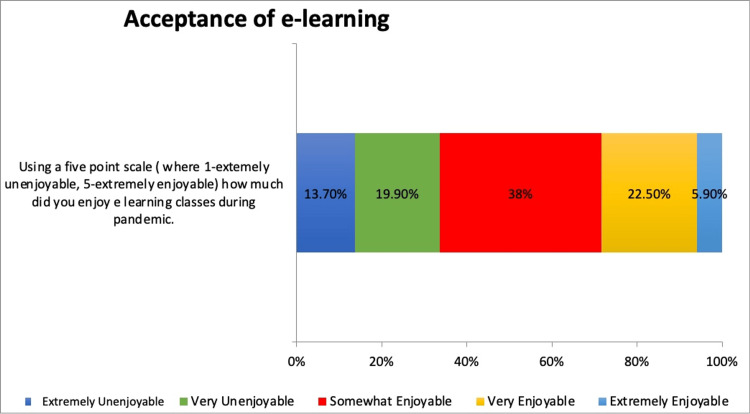
Acceptance of e-learning during pandemic

The majority of the students perceived e-learning as somewhat enjoyable, whereas the remaining respondents considered it very enjoyable. However, some of the respondents considered it very unenjoyable (Figure 5).

## Discussion

The research was conducted to evaluate how e-learning contributes to the improvement of knowledge, clinical skills, and social competencies. Students’ involvement and acceptance were also examined. To our understanding, this study represents the pioneering endeavor of its nature conducted among healthcare students within a tertiary care hospital setting in Multan, Pakistan. The global COVID-19 pandemic has presented significant challenges to educational institutions worldwide, notably due to the transition from traditional face-to-face instructional sessions to online teaching modalities [20].

Digital learning stands as a universally effective tool for both teaching and learning. Before the pandemic, it was not popular in most developing countries such as Pakistan because faculty and students were not familiar with digital system innovations. Lack of training and guidance were the probable causes for this lack of familiarity, even though substantial resources were allocated for obtaining electronic communication equipment and shifting Pakistan toward digitalization [21].

Medical students in Pakistan were more inclined toward traditional face-to-face learning before COVID-19 [22]. However, after the pandemic began, Pakistani students were asked to shift toward e-learning. Initially, they rated it as very low because of its limitations for use in clerkships and labs/clinicals. This finding was somewhat aligned with those in many other countries such as China, Singapore, and Malaysia [23].

The major advantages of online learning are the availability of resource materials and instant guidance from facilitators. Students have greater communication opportunities and can foster self-paced learning through the learner-centered approach. E-learning is valued because it provides distance learning opportunities, reducing the cost of learning and allowing students to learn in a relaxing and comfortable environment. A study conducted in the UAE and Poland supported digitalization in this context [24,25].

In a developing country like Pakistan, it is difficult to hinge upon online teaching and learning because of technical flaws in the country’s digital learning system. Most respondents listed technical issues and reduced patient interaction as the major challenges facing e-learning, in line with a similar study on Palestinian students [26].^ ^However virtual patients and telemedicine were seen as effective substitutes. Virtual patients can simulate numerous real-life schemas, preparing students for clinical encounters. As William Osler stated, “He who studies medicine without books, sails an uncharted sea, but he who studies medicine without patients, does not go to sea at all” [27]. Increased patient exposure in face-to-face sessions helped fill the gap of reduced patient interactions during the pandemic.

Interestingly, the majority of the respondents claimed that they already had an online learning experience. This result was in contrast to that of another study conducted in Poland. In that study, the majority of the respondents had no online learning experience. The current study was conducted during the fourth wave of the COVID-19 pandemic. In most colleges in Pakistan, online teaching and learning systems were already running at the institutional level. The current study revealed that a very small percentage of students believed they had remarkable technical expertise. In comparison, the majority of the students in Poland believed they had remarkable technical expertise. In the majority of developing countries, technology advancement is slow and most students face difficulties due to persistent connectivity issues [28].

This study compared face-to-face learning with online learning, revealing that a majority of respondents regarded traditional teaching as more effective in enhancing knowledge, skills, and social competencies. These findings resonate with prior research on remote communication versus face-to-face interaction in clinical dental education and students' perceptions of online learning amidst the pandemic [29]. However, contrary to the findings of those studies, the present study indicated that the majority of respondents deemed face-to-face sessions more effective than online teaching in enhancing knowledge. Blended learning can be an effective substitute for both learning types because it gives students the flexibility to set their own schedules and the opportunity to clarify difficult concepts in the classroom, make connections, and organize their knowledge. The ratio of traditional teaching to online teaching should be carefully planned to produce maximum results [30].

Despite the widespread popularity of online learning during the COVID-19 pandemic, the majority of respondents in this study reported being more active during face-to-face sessions and less active during online learning. The following are some possible reasons for this phenomenon:

First, students were more inclined toward traditional teaching. Furthermore, there was a deficiency in technical platforms to adequately support online teaching and learning requirements. Training on online management systems and interactive activities during e-learning was also not provided to students and teachers [21].

Interestingly, most of the respondents found online learning enjoyable. This finding was in line with a study on acceptance of e-learning during COVID-19. In that study, respondents considered online teaching to be a potent alternative to traditional teaching. E-learning was found to be a potential solution for sustaining educational activities during an institutional lockdown [27].

The recommendations that are concluded from the study are training workshops for online teaching and learning (awareness of technology, gadgets, and their educational uses for the faculty and students) can be beneficial in enhancing the skills of the students and may improve the effectiveness of online sessions [28]. Future research on the feasibility of clinical training with the help of virtual patient simulation using standardized computer software allows the incitement of real-life scenarios for the incorporation of important skills like clinical reasoning. ​​Keeping potential issues in mind future research on the Digitalization of patient records so that the students may easily access patient records and videos anytime for learning purposes without breaching the confidentiality of the patients. Future research on the use of artificial intelligence-based learning management systems should be promoted in the institutions delivering online classes. Artificial intelligence-based learning management systems may utilize surveys during online classes to categorize students according to their learning requirements (auditory, visual, and text-related) and provide them with learning content that conforms to their learning style.

The limitations of the study are, that it was conducted in a single institution; thus, institutional bias was a possible factor. Moreover, questionnaires were administered to students through an online platform, possibly resulting in a lower number of responses. Hence, it's important to note that the findings of this study may not be universally applicable or representative of the entire population.

## Conclusions

This study concluded that e-learning was advantageous to the learners on the grounds of flexibility of the study location and ease of access to the study materials. Most of the respondents claimed to have had a previous e-learning experience and were satisfied with the sessions. However, when contrasted with online teaching sessions, participants perceived traditional face-to-face teaching as more effective in enhancing knowledge and improving skills and social competencies. Moreover, the students felt more engaged and active in physical classrooms. Hence, e-learning should not be used exclusively for teaching and is not a preferable alternative to traditional face-to-face teaching sessions due to its critical drawbacks such as technical issues during an e-learning session and reduced patient exposure during clinical teaching sessions. Consequently, this supports the fact that clinical learning and teaching environments cannot be recreated entirely during an e-learning session. The study concluded that while e-learning offers flexibility, traditional face-to-face teaching is deemed more effective for skill development and social interaction. Hence, e-learning should complement rather than replace traditional methods due to limitations in replicating clinical environments.

## Appendices

Questionnaire 

I.     **Basic demographics:**

1.    **What is your current age (in years)?**

__________________________

2.   ** What is your gender?**

☐ Male                 

☐ Female

3.    **In which health-related facility of Bakhtawar Amin do you study?**

☐ Dental College

☐ Medical College

☐ Physiotherapy College

☐ Allied College

4.   **What year of medical studies are you in?**

☐ 1st Year          ☐ 2nd Year         ☐ 3rd Year        ☐ 4th Year       ☐ 5th Year 

5.   **How would you describe your IT skills?**

☐ High          ☐ Moderate          ☐ Low

6.   **Have you ever participated in any type of e learning before the pandemic?**

☐ Yes                              ☐ No

II.   **Advantages and disadvantages of E-learning:**

7.   **What are the advantages of E-learning?**

(Pick all that apply)

☐ Access to online materials                   ☐ Classes interactivity

☐ Ability to record a meeting                ☐ Learning on your own pace 

☐ Comfortable surrounding                   ☐ Ability to stay at home

8.   **What are the disadvantages of E-Learning?**

(Pick all that apply)

☐ Reduced interaction with the teacher              ☐ Technical problems

☐ Lack of interactions with patients                   ☐ Lack of self-discipline

☐ Poor learning conditions at home                   ☐ Social isolation

III.  **Comparison between face-to-face and E-Learning in terms of ability to master domains like knowledge, clinical skills and social competencies:**

Using a 5-point likert scale to rate the questions:

1-Extremely Ineffective

2-Ineffective

3-Uncertain

4-Effective

5-Extremely effective

9.   Effectiveness of E-learning in terms of increasing knowledge skills:

☐1                    ☐2                      ☐3                     ☐ 4                   ☐ 5

10. Effectiveness of E-learning in terms of increasing clinical skills:

☐1                    ☐2                      ☐3                     ☐ 4                   ☐ 5

11. Effectiveness of E-learning in terms of increasing social competencies:

☐1                    ☐2                      ☐3                     ☐ 4                   ☐ 5

12. Effectiveness of face-to-face learning in terms of increasing knowledge:

☐1                    ☐2                      ☐3                     ☐ 4                   ☐ 5

13. Effectiveness of face-to-face learning in terms of increasing clinical skills:

☐1                    ☐2                      ☐3                     ☐ 4                   ☐ 5

14. Effectiveness of face-to-face learning in terms of increasing social competencies:

☐1                    ☐2                      ☐3                     ☐ 4                   ☐ 5

IV.          Acceptance of E-Learning:

**Using a 5-point Likert scale to rate the questions:**

1-Extremely Unenjoyable

2-Very Unenjoyable

3-Somewhat Enjoyable

4-Very Enjoyable

5-Extremely Enjoyable

15. How much did you enjoy E-Learning classes during the pandemic?

☐1                   ☐2                      ☐3                     ☐ 4                   ☐ 5
